# Evaluation of suitable reference genes for gene expression studies in bovine muscular tissue

**DOI:** 10.1186/1471-2199-9-79

**Published:** 2008-09-11

**Authors:** Raquel Pérez, Isabel Tupac-Yupanqui, Susana Dunner

**Affiliations:** 1 Dpt. Animal Production, Veterinary Faculty, University Complutense of Madrid, 28040 Madrid, Spain

## Abstract

**Background:**

Real-time reverse transcriptase quantitative polymerase chain reaction (real-time RTqPCR) is a technique used to measure mRNA species copy number as a way to determine key genes involved in different biological processes. However, the expression level of these key genes may vary among tissues or cells not only as a consequence of differential expression but also due to different factors, including choice of reference genes to normalize the expression levels of the target genes; thus the selection of reference genes is critical for expression studies. For this purpose, ten candidate reference genes were investigated in bovine muscular tissue.

**Results:**

The value of stability of ten candidate reference genes included in three groups was estimated: the so called 'classical housekeeping' genes (18S, GAPDH and ACTB), a second set of genes used in expression studies conducted on other tissues (B2M, RPII, UBC and HMBS) and a third set of novel genes (SF3A1, EEF1A2 and CASC3). Three different statistical algorithms were used to rank the genes by their stability measures as produced by geNorm, NormFinder and Bestkeeper. The three methods tend to agree on the most stably expressed genes and the least in muscular tissue. EEF1A2 and HMBS followed by SF3A1, ACTB, and CASC3 can be considered as stable reference genes, and B2M, RPII, UBC and GAPDH would not be appropriate. Although the rRNA-18S stability measure seems to be within the range of acceptance, its use is not recommended because its synthesis regulation is not representative of mRNA levels.

**Conclusion:**

Based on geNorm algorithm, we propose the use of three genes SF3A1, EEF1A2 and HMBS as references for normalization of real-time RTqPCR in muscle expression studies.

## Background

In the last few years, Real-time reverse transcriptase quantitative polymerase chain reaction (Real-time RTqPCR) has been successfully used to measure mRNA species copy number as a way to determine key genes involved in different biological processes: disease, economic traits, etc. [e.g. [1-4]]. This technique shows a high sensitivity over a wide range of transcript expression levels and enables high throughput capabilities [5]. Nevertheless, it is subject to substantial technical variability in expression measures due to different factors such as type and quality of samples [6,7], starting cell number, RNA extraction and reverse transcription methods [8-10]. Moreover, the biological interpretations of expression results critically depend on normalization of transcript signals to mRNA standards before statistical evaluation, which will allow the control of the variability produced by all the mentioned factors [11]. Normalization of the expression levels of the target genes is performed through reference genes [12,10] also called housekeeping [13], which are internal endogenous controls that should be constitutively expressed in a tissue, across samples and treatments. Misinterpretation of data occurs when expression measures are erroneously normalized to a subset of mRNAs that are subject to strong regulation [14,15]. The correct reference genes can be selected by evaluating data from Real-time RTqPCR with statistical algorithms such as geNorm [16], Bestkeeper [17] or Normfinder [18]. Common reference genes for normalization of qRT-PCR data in skeletal muscle include ACTB, β2-microglobulin, GAPDH, PPIA, and 18S and 28S rRNAs [19-21]. Most studies use only one reference gene, generally 18S rRNA, ACTB or GAPDH [see [20,22,23], respectively], and more rarely B2M or PPIA [see [24,25] respectively]; however, the analysis of the stability of these genes in muscle shows contradictory conclusions [19,26,27]. Erkens and co-workers [28] checked 10 different reference genes for pig muscle expression studies, proposing ACTB, TBP and TOP2B as good references in measures by Real-time RTqPCR to contrast between different muscle fibers and between muscle and adipose tissue. Also Nygard et al. [29] select high quality reference genes for real-time qPCR data interpretation in muscle tissue and others.

In the present study, the expression stability and level of ten candidate reference genes is measured with the aim of creating a set of genes which can be used in bovine skeletal muscle tissue for normalization of mRNA measures by Real-time RTqPCR. For this purpose we evaluate a set of "classical housekeeping" genes (18S, GAPDH and ACTB), a second set of genes used in expression studies conducted on other tissues (B2M, RPII, UBC and HMBS) and a third set of other genes (SF3A1, EEF1A2 and CASC3) on samples of *Longissimus dorsi *for which fatty acid profiles have been measured, in an effort to avoid misinterpretation of expression data produced in transcription studies of bovine skeletal muscle samples.

## Methods

### • RNA source, total RNA extraction and cDNA synthesis

A total of 120 bovine individuals were measured for long chain omega 3 fatty acids [%LCω3 = % eicosapentaenoic acid (20:5, ω3; EPA) + % docosapentaenoic acid (22:5, ω3; DPA) + % docosahexaenoic acid (22:6, ω3; DHA)]. Ten of these individuals showing the highest and ten showing the lowest percentages (differences p < 0.001) for this phenotype were selected. For each, a sample of 25 mg from *Longissimus dorsi *taken shortly after slaughtering was homogenized and RNA was extracted using commercial spin-columns (RNeasy^® ^Fibrous Tissue Mini Kit, QIAGEN), yielding around 10–20 μg of total RNA protected against RNase degradation with RNA *secure*™ Reagent 1× (Ambion).

Two μl of total RNA were used to produce a retro-transcription reaction using an iScript™ cDNA Synthesis Kit (Bio-Rad), following the manufacturer's recommendations. To perform gene testing, part of the reaction was diluted 1/10 and part was pooled and serial diluted to construct the standard curves; all the aliquots were stored at -70°C until use. The quality and concentration of total RNA representing each sample was assessed by conventional agarose electrophoresis and through absorbance measurements (ratio 260/280 ≥ 2). Intact 28S and 18S rRNA subunit were observed on the gel indicating minimal degradation of the RNA.

### • Selection of genes and primer design

Known sequences were used to design primers for eight genes (Table 1) using *primer 3 *[30], preventing possible secondary structures with QIAGEN Oligo Toolkit  and Dinamelt server [31], and ensuring the specificity of the sequence by BLAST [32,33]. Two primer pair sequences were defined by Chitko-McKown and co-workers [34] and Goossens et al. [35]

**Table 1:** Selected candidate reference genes used in the Real-time RTqPCR assay indicating name, GenBank accession number or reference, function, annealing temperature (T_a_), PCR efficiency, regression coefficient and primers used for the expression study.

Gene	Full gene name	GenBank accession number or reference	Function	T_a_	PCR efficiency	Regression coefficient (r^2^)	Forward primer	Reverse primer
18S	18S ribosomal RNA	DQ222453.1	Ribosomal eukaryotic small subunit	57°C	101.1%	0.983	CGGCTACCACATCCTATGAA	TGGAGCTGGAATTACCGCGG
ACTB	β-actin	Goossens et al., 2005	Cytosqueletal structural protein	59°C	97.0%	0.982	CCTCACGGAACGTGGTTACA	TCCTTGATGTCACGCACAATTT
B2M	β-2-microglobulin	NM_173893.2	Beta-chain of major histocompatibility complex class I molecules	59°C	99.1%	0.981	AGTAAGCCGCAGTGGAGGT	CGCAAAACACCCTGAAGACT
CASC3	cancer susceptibility candidate 3	XM_610173.2	Linked to development of breast cancer	57°C	97.8%	0.95	TACATCCCCACCAGACACC	GGAGCAGAAAAGTAAGTAGGAGCA
EEF1A2	eukaryotic translation elongation factor 1 alpha 2	BC108110.1	Translation elongation factor activity	59°C	99.3%	0.972	GCAGCCATTGTGGAGATG	ACTTGCCCGCCTTCTGTG
GAPDH	glyceraldehyde-3-phosphate dehydrogenase	NM_001034034.1	Oxidoreductase in glycolysis and gluconeogenesis	59°C	99.6%	0.979	TGCACCACCAACTGCTTGGC	GGCATGGACGGTGGTCATGAG
HMBS	hydroxymethylbilane synthase	BC112573.1	Heme synthesis, porphyrin metabolism	59°C	95.4%	0.988	CTTTGGAGAGGAATGAAGTGG	AATGGTGAAGCCAGGAGGAA
RPII	polymerase (RNA) II (DNA directed) polypeptide A (220 kD)	NM_000937.2	DNA-directed RNA polymerase II subunit	59°C	103.6%	0.988	ACCCACTAGCCCCACCTACT	GCTCACCCTCAGTTCTCGTC
SF3A1	splicing factor 3 subunit 1	XM_878187.1	Structural component of the splicing system	57°C	95.0%	0.981	GCGGGAGGAAGAAGTAGGAG	TCAGCAAGAGGGACACAAA
UBC	Ubiquitin C	Chitko-McKown et al, 2004	Protein degradation	59°C	103.4%	0.976	TCCCTACCTGCATCATGTGC	GGAATTTGGGCCAGTGCTC

### • Real-time RTqPCR

Real-time RTqPCR reactions were performed in an iCycler IQ Real-Time PCR Detection System (Bio-Rad) and a master mix was prepared using Dynamo™ HS SYBR^® ^Green qPCR Kit (Finnzymes), 0.4 mM of each primer and 2.7 μl of 1/10 diluted RT (regardless of initial concentration) in 15 μl reaction volumes. After the selection of the most adequate annealing temperature, standard curves and no-template controls were produced in triplicate for each gene, together with the sample assays. The following experimental run protocol was used: quantification program consisting of 45 cycles of 95°C for 25 sec, 10s at annealing temperature and 15 s at 72°C, ending with a melting program ranging from 68°C to 95°C with a heating rate of 0.1°C/10 sec and continuous fluorescence measurement.

The results were exported from the iCycler IQ Real-Time PCR Detection System into Microsoft Excel files for further analysis.

### • Data Analysis

Real-time RTqPCR data were exported into an Excel datasheet (Microsoft Excel 2003) and analyzed using three separate reference gene stability analysis software packages; geNorm [16], Bestkeeper^© ^[17] and NormFinder [18]. The three methods generate a measure of reference gene stability, which can be used to rank the reference genes; GeNorm generates an M value for each gene which is arbitrarily suggested to be lower than 1.5 (with a lower value indicating increased gene stability across samples), and a pairwise stability measure to determine the benefit of adding extra reference genes for the normalization process (again with a lower value indicating greater stability of the normalization factor). An arbitrary cut off value of 0.15 indicates acceptable stability of the reference gene combination. Similarly, NormFinder generates a stability measure of which a lower value indicates increased stability in gene expression and groups samples to allow direct estimation of expression variation, ranking genes according to the similarity of their expression profiles by using a model-based approach. Bestkeeper^© ^generates a pairwise correlation co-efficient between each gene and the Bestkeeper index (the geometric mean of the threshold cycle values of all the reference genes grouped together).

## Results and discussion

When faced with any tissue expression experiment, the lack of really stable reference genes makes misinterpretation of results a high risk [11]. It is considered necessary to have a list of genes which will enable satisfactory normalization of the expression data of the experiment and permit biological conclusions. There are many different ways to test the aptitude of a control gene for normalization in an experimental design and they have been compared with contradictory results [e.g. [18] or [36]]; although all give an order of candidate reference genes on the basis of the relative expression stabilities through mathematical evaluation of expression data, in our opinion geNorm [16] seems the best option as it proposes the number of reference genes necessary for accurate normalization, helping to choose the most stable genes by expressing a stability measure (M), which is the average pairwise variation for a given gene compared to all the other tested genes. Stepwise exclusion of the gene with the highest M value allows ranking of the tested genes according to their expression stability. Bestkeeper [17] and Normfinder [18] also rank the candidate reference genes and Normfinder is able to identify the single gene with the most stable expression, whereas geNorm detects the two genes whose expression ratios show least variation from those of the other genes tested. On the other hand, Bestkeeper does not restart pair-wise correlation after one gene is eliminated; nevertheless, results with geNorm are quite similar. Results of the three algorithms comparison and identification of the set of reference genes that offers reliable results for Real-time RTqPCR data normalization for use in gene expression studies involving muscular tissue from bovine individuals are listed in Table 2.

**Table 2:** Expression stability values of the candidate housekeeping genes calculated by the geNorm, Normfinder and Bestkeeper algorithms (ranking in parentheses).

Gene	Stability Value GeNorm	Stability Value Normfinder	Stability Value Bestkeeper
EEF1A2	0.63 (1)	0.23 (1)	0.86 (2)
SF3A1	0.63 (2)	0.48 (4)	0.91 (5)
HMBS	0.78 (3)	0.39 (2)	0.61 (1)
ACTB	0.83 (4)	0.48 (4)	1.19 (8)
CASC3	0.90 (5)	0.41 (3)	0.89 (4)
UBC	0.95 (6)	0.68 (8)	1.31 (9)
B2M	0.98 (7)	0.61 (7)	1.17 (7)
RPII	1.15 (8)	0.51 (6)	0.99 (6)
18S	1.22 (9)	0.76 (9)	0.86 (2)
GAPDH	1.62 (10)	1.55 (10)	3.14 (10)

The need to validate a collection of reference genes in every tissue and between different treatments to ensure correct normalization has led us to validate a list of genes as reference for studies by Real-time RTqPCR of skeletal muscle fatty acid metabolism. Information on reference genes for use in expression normalization of samples from skeletal muscle tissue is scarce, although different papers address the same problem in other tissues [e.g. [29,37]]. The ten different candidate genes were checked for different reasons: GAPDH, ACTB and 18S rRNA are used as single control genes in more then 90% of the published expression studies [21] and are specifically used as reference genes in *Longissimus dorsi *in the pig [28]. However, it has been reported that ACTB is most relevant for high abundant transcript [29] and together with GAPDH, it fluctuates dramatically [21] and should be rejected. Although the 18S rRNA measure of stability seems to be within the range of acceptance (when using geNorm but also for Bestkeeper), it has repeatedly been documented that it is not a good control gene [see e.g. [38,15,39]], as its synthesis regulation is not representative of mRNA levels [40].

B2M, RPII and UBC are frequently used for mRNA measures as reference genes in other tissues but not in skeletal muscle [41]. We added SF3A1, EEF1A2, HMBS and CASC3 as genes with validated stability in several cellular classes [16,42,43], but not in mammalian skeletal muscle. All the genes chosen belong to different functional classes to avoid co-variation between them.

Melting curves generated (not shown) ensure the correct amplification of all genes tested in this work when using the primers shown in Table 1, with PCR amplification efficiency values near to 100% [8,9]; correlation coefficients (r^2^) between the logarithm of the cDNA starting quantity and the Ct were, at least, 0.95 for all genes (Table 2). Negative controls lacking template show no amplification or a very late exponential growth and, in this case, the melting curve reveals clearly identified negligible peaks.

**Figure 1:**
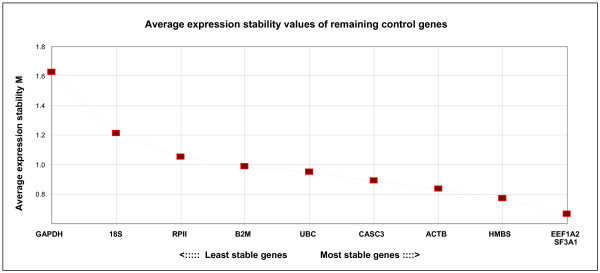
**Gene expression stability of candidate reference genes**. Gene expression stability of candidate reference genes in bovine muscular tissue analyzed by the geNorm program which proceeds to the stepwise exclusion of the genes whose relative expression levels are more variable among tissue samples. Threshold for eliminating a gene as unstable was M ≥ 1.5. Lower values of M correspond to the most stable genes, hence the most suitable for normalization.

GeNorm calculation of the internal control gene-stability measure (M) ranks our gene expression variations (Figure 1), SF3A1 and EEF1A2 being the most stable with an M value around 0.66. When ranking the reference genes by different methods (Table 2), we found that EEF1A2 appears as the best reference gene both in geNorm and Normfinder. Genes HMBS, ACTB, CASC3 and B2M can be accepted as stable with M values between 0.7 – 1, and to a lesser extent RPII and 18S with M values below 1.5.

Use of GAPDH as a housekeeping gene would not be appropriate, as it appears to be regulated in muscle tissue showing an M value of 1.63 and is thus not recommended as a reference gene. Actually this gene is ranked as the worst with all algorithms used here and the inadequate use of this gene has also been documented in other studies [43,28]. Our assessment is that GAPDH, 18S and RPII are not able to make conclusions obtained by expression measures normalized with them, as these genes show expression differences between the two sets of samples.

**Figure 2:**
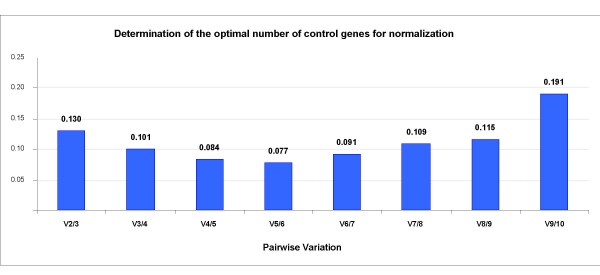
**Evaluation of the optimum number of reference genes according to the geNorm software**. The magnitude of the change in the normalization factor after the inclusion of an additional reference gene reflects the improvement obtained. V*i*/*i+1 *represent the models being compared: those with *i *and *i+1 *reference genes.

As regards the number of genes to be used in expression studies, de Jonge et al. [21] report that no single gene qualifies as a 'real' housekeeping gene. The calculation of V values by geNorm for the proposed genes (Figure 2) is useful for deciding their optimal number to be used in an expression study [16]; pairwise variation between samples is reduced by the inclusion of additional reference genes and thus indicates the number of genes required to achieve an arbitrarily selected threshold of reference gene stability; a recommended cut-off value of 0.15 shows genes SF3A1 and EEF1A2 as having good stability in relative quantification. If HMBS, ACTB and/or CASC3 are added, a more accurate normalization can be performed, but the use of more than three control genes is unnecessary for the analyses [28].

## Conclusion

Using the geometric mean of the three most stable genes listed, we conclude that SF3A1, EEF1A2 and HMBS would lead to powerful results in bovine skeletal muscle tissue. These genes have already shown stability in breast cancer [42], salmon muscle [43] and human fibroblasts [16], and now show to be stable in mammalian skeletal muscle transcriptome studies.

## Authors' contributions

RP and IT carried out the qPCR experiments, participated in the election of genes and drafted the manuscript. RP and SD participated in the design of the study and the statistical analysis. SD contributed to drafting the manuscript and supervised the process. All authors read and approved the final manuscript.
